# Methods for Discerning the Impact of Mucus on Host Defenses Against Viral Infection

**DOI:** 10.1002/cpz1.70201

**Published:** 2025-09-19

**Authors:** Maria Corkran, Allison Boboltz, Gregg A. Duncan, Margaret A. Scull

**Affiliations:** ^1^ Department of Cell Biology & Molecular Genetics, Maryland Pathogen Research Institute University of Maryland College Park Maryland; ^2^ Fischell Department of Bioengineering University of Maryland College Park Maryland; ^3^ These authors contributed equally to this work

**Keywords:** airway clearance, mucus, respiratory viruses

## Abstract

Mucus is an important component of airway host defenses that acts by enabling the trapping and clearance of infectious materials such as bacteria and viruses. It can be difficult, however, to design experiments that independently determine the extent to which mucus contributes to innate barrier functions in the lung. Here, we provide detailed protocols to collect mucus from human airway epithelial cultures and evaluate how the properties of mucus impact mucociliary transport and protection from viral infection. We include recommended test parameters depending on the specific research question as it relates to respiratory infectious diseases. © 2025 The Author(s). Current Protocols published by Wiley Periodicals LLC.

**Basic Protocol 1**: Analysis of mucociliary transport and ciliary beat frequency in HAE cultures

**Basic Protocol 2**: Collection of mucus from HAE cultures

**Basic Protocol 3**: Transplantation of mucus to HAE cultures and infection with virus

## INTRODUCTION

The purpose of these protocols is to evaluate the impact of diverse mucus environments on airway clearance function and susceptibility to infection in an *in vitro* model of human airway epithelium (HAE). HAE cultures grown at an air‐liquid interface (ALI) recapitulate critical aspects of the epithelium *in vivo*, including the diverse cell types present. Further, HAE ALI cultures give rise to the periciliary layer where cilia are provided a well‐hydrated environment, enabling a metachronal ciliary beat that brings about the movement of the overlying secreted mucus barrier, and thus modeling mucociliary transport (MCT) (Button et al., 2012). An important advantage of this system is the ability to easily access and experimentally manipulate this extracellular microenvironment in an *in vitro* setting, facilitating studies on the first line of defense in the airways: the secreted mucus barrier.

Historically, modeling the mucus barrier *in vitro* has involved reconstitution of isolated and purified mucins from bovine or porcine tissues (Rehm & Moradali, 2021). Although these materials are relatively abundant and yield reproducible gels, the procedures for mucin purification have been shown to change key physicochemical properties, such as their degree of glycosylation and polymerization, leading to viscoelastic properties that do not resemble native mucus gels (Kocevar‐Nared et al., 1997; Marczynski et al., 2021). The ability of the HAE culture system to produce native mucus that can be harvested directly highlights its value as a source of human mucus that may better recapitulate the properties of the mucosal barrier within human airways. Because these cultures are generated in‐lab from basal cells, they can also be integrated into workflows that enable manipulation of host targets (e.g., by knockdown). The development of an immortalized airway basal cell line originating from a healthy donor, the BCi‐NS1.1 cell line, allows for additional passaging and selection of modified cells prior to differentiation, further supporting investigation of the role of specific proteins in a physiologically relevant model (Walters et al., 2013). For instance, our group has investigated the contribution of each of the two major secreted airway mucins to mucociliary transport by CRISPR/Cas9‐mediated depletion of each mucin individually in BCi‐NS1.1 cells, and subsequent generation of HAE cultures that produce human airway mucus enriched for specific mucin proteins (Song et al., 2022). Alternatively, synthetic polyethylene glycol (PEG) hydrogels established with a crosslinker reagent to form disulfide bonds have been used to imitate mucus architecture. Since these synthetic hydrogels lack the proteins and glycans typically present in mucus, they represent a useful tool to specifically interrogate the contribution of mucus network structure to its barrier function. The use of such hydrogels to coat the apical surface of differentiated HAE cultures in place of the native mucus has also been reported (Kaler et al., 2025).

Since the advent of HAE ALI cultures in the 1980s, insights into the human airway microenvironment have advanced significantly (Jorissen et al., 1989; Whitcutt et al., 1988; Wu et al., 1985). Still, much work remains to be done to fully understand mucus as a biochemical, semi‐permeable extracellular barrier against specific inhaled pathogens. Here, we describe a method for characterizing MCT properties and probing the specific physical barrier function of mucus in this physiologically relevant system. This method employs real‐time imaging of the cultures followed by a custom‐written image‐processing algorithm in MATLAB. By tracking fluorescent microspheres added apically to the culture surface, as well as imaging in brightfield to capture the local pixel intensity maxima over time, the mucus transport rate and ciliary beat frequency (CBF) can be determined. Following characterization of MCT and CBF, specific barrier functions of the mucus can be assessed. In this case, harvesting native mucus from HAE cultures and then transplanting this mucus back onto healthy cultures enables the researcher to control the volume (i.e., thickness) of the mucus sample. Further, by transplanting synthetic (glycan‐free) PEG‐based hydrogels with matched network structure alongside native mucus, insights into the potential contributions of adhesive and steric interactions on mucus barrier function can be gained.

Basic Protocol 1 describes how to analyze fully differentiated HAE cultures for MCT and CBF using video tracking of particles on the apical surface. Basic Protocol 2 describes how to wash the apical surface of HAE cultures to remove mucus. This is important both for regular maintenance of differentiated HAE cultures and to collect and concentrate mucus for transplantation. Basic Protocol 3 describes how to (1) transplant mucus to an HAE culture that has been washed to remove native mucus, (2) allow the mucus to spread and form a barrier that is ready for investigation, (3) infect the transplanted culture with virus, and (4) prepare apical washes and fixed cultures to assess the level of infection. The preparation of a hydrogel barrier is not presented, but the analysis of such a system is the same as described here.

*NOTE*: All cell culture work should be performed under sterile conditions in a certified biological safety cabinet (BSC) following BSL2 protocols. All institutional regulations for research using human cells and viruses must be followed.

## Basic Protocol 1 ANALYSIS OF MUCOCILIARY TRANSPORT AND CILIARY BEAT FREQUENCY IN HAE CULTURES

HAE cultures are generated by seeding basal airway epithelial cells on porous Transwell membranes, growing the cells to confluence, and maintaining them at an air‐liquid interface to promote differentiation. Differentiation into a pseudostratified epithelium that models MCT takes ∼4 weeks. A detailed, step‐wise guide for the growth and maintenance of HAE cultures has been previously reported (Gagliardi et al., 2022). Therefore, we focus here on HAE culture analysis post‐differentiation.

**Figure 1:**
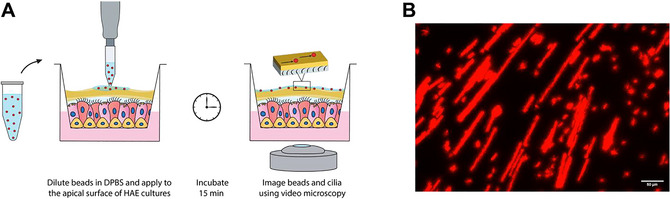
(**A**) Schematic overview of MCT workflow. (**B**) Maximum intensity *z* projection of microspheres undergoing MCT over the mucosal surface of an untreated BCi‐NS1.1 culture over the course of a 20‐s video using the procedure outlined below. Scale bar, 50 µm.

MCT is a vital function of the pulmonary epithelium that is often disrupted in both infectious and non‐infectious respiratory diseases. The velocity of mucus transport by HAE cell lines and primary cells grown at an air‐liquid interface can be measured by tracking the motion of particles on the mucosal surface using particle image velocimetry. The following is a protocol that uses video microscopy to track the transport of fluorescent micro‐scale particles (microspheres) across the apical surface of HAE cells at an air‐liquid interface, which can later be analyzed to quantify the velocity of MCT (Boboltz et al., 2023; Iverson et al., 2022; Song et al., 2021; Song et al., 2022). See Figure 1 for an overview of the MCT workflow.

Changes in MCT may be the result of altered ciliary beating. Thus, we also describe the collection of videos of the apical surface of HAE cultures using brightfield microscopy, which can later be analyzed to quantify CBF.

### Materials

70% (v/v) isopropanol or ethanol
Fully differentiated human airway epithelial (HAE) cultures at the air‐liquid interface in 12‐well, 12‐mm Transwells (cell growth area = 1.12 cm^2^; Corning, cat. no. 3460)
Sterile 1× Dulbecco's phosphate‐buffered saline (DPBS), no calcium, no magnesium (Gibco, cat. no. 14190144)
2.0‐µm red carboxylate–modified microspheres (FluoSpheres, Invitrogen, cat. no. F8826)

Biological safety cabinet (BSC) certified for BSL2 work
37°C water bath
Incubator (37°C, 5% CO_2_)
Aspirating pipettes and vacuum
Single‐channel pipettes and sterile barrier tips for volumes of 1‐1000 µl
Bath sonicator (Branson 2800)
Microscope with camera, for widefield fluorescence and brightfield microscopy, with 10× objective and stage insert compatible with multiwell plates (Zeiss LSM 800 with Axiocam 702 mono camera, connected to Zen microscopy software)
MATLAB software with the following scripts installed and open: TrackerDriver_20180312_MCC.m, VelocityCalculator_1toend.m, CiliaBeating.m
Scientific graphing software (e.g., GraphPad Prism)
Fiji software (ImageJ; www.imagej.net/ij; Schindelin et al., 2012)

#### Apply microspheres to HAE cultures

1 Prepare the BSC for sterile cell culture work, wiping any materials brought into the hood with 70% isopropanol or ethanol.
2 In the BSC, remove mucus and cell debris from the apical surface of each HAE culture (12‐mm Transwell) to be analyzed. Add 250 µl DPBS (pre‐warmed in a 37°C water bath) onto the apical surface of each culture using a 1000‐µl pipette and return plate to the incubator for 30 min.
  We recommend washing the cultures 24 hr prior to MCT analysis to remove thick mucus deposits that may inhibit MCT, while still allowing time for the HAE cultures to regenerate a secreted mucus layer.
3 Remove DPBS using an aspirating pipette attached to a vacuum.
  When aspirating the DPBS, gently tip the plate forward with one hand so the DPBS pools toward the edge of the apical compartment of the Transwell but does not leak out into the basolateral compartment. Ensure the aspirating pipette does not touch the cells. In some cases, it may be beneficial to fit a 200‐µl non‐barrier tip to the end of the aspirating pipette to facilitate DPBS removal.
4 Sonicate microspheres in a bath sonicator for 10 min to disrupt aggregates.
5 Place microspheres in the BSC and dilute 1:3000 in sterile DPBS. Vortex briefly.
6 Add 20 µl diluted microspheres to the surface of each culture and return plate to the incubator for 15 min.
  It is usually easiest to work in batches of two to three cultures. If working in batches, it will be necessary to transfer the batch of HAE cultures to a separate 12‐well plate using tweezers and add 1 ml cell culture medium to the basolateral compartment before applying the microspheres, then return the remainder of the HAE cultures (in the plate without microspheres) to the incubator until ready for the next batch. This method minimizes the amount of time the cultures spend outside of the incubator and helps avoid large alterations in MCT or CBF caused by different atmospheric conditions. If using a microscope incubation chamber set to 37°C and 5% CO_2_, or using a small number of HAE cultures in the experiment, the batch method may not be necessary.
  If using a cell culture insert with a smaller membrane surface area (e.g., 24‐well, 6.5‐mm Transwells with a cell growth area of 0.33 cm^2^), the volume of microspheres applied should be reduced accordingly.
7 During incubation, set up the microscope with the following settings: widefield mode, 20‐s videos, 100‐ms exposure time, 10× objective.
8 After 15 min, take the plate out of the incubator and place it on the microscope stage.
  Cells may be imaged directly in the tissue culture plate with medium underneath.

#### Collect MCT data

9 Select the excitation/emission wavelength settings corresponding to the color of the microspheres used. Adjust the 10× objective until the microspheres are in focus.
  The microspheres will be primarily on one plane, as they sit atop the mucosal surface of the cultures. The large 2‐µm size prevents diffusion/Brownian motion within the pores of the mucus gel. If smaller microspheres are used, they will likely appear to “vibrate” as they undergo Brownian motion and diffuse within the mucus layer.
  It is best to avoid imaging areas that are extremely dense with microspheres (i.e., where individual microspheres are indistinguishable from each other). If the microspheres are too bright to see any definition at 10× magnification, lower the exposure time (this can be accounted for in the analysis) and/or laser intensity.
  The microspheres should be moving, but this can be difficult to detect by eye if transport is slow. In this case, take a test video and play it back at a higher speed to determine if the microspheres are moving.
  Focusing on the microspheres may be challenging depending on the microscope and brand of cell culture insert used. This is usually because the cell culture insert is sitting too high above the bottom of the multiwell plate for the objective to focus. In this case, place the multiwell plate back in the BSC. Carefully remove the cell culture insert with microspheres from the 12‐well plate using tweezers, keeping the cultures upright, and place in a well of a sterile, empty 6‐well plate so the bottom of the insert membrane is flush with the bottom of the plate. Once transfer is complete, place the lid back on the 12‐well plate and return these cultures to the incubator. Close the lid of the 6‐well plate before removing it from the BSC for imaging on the microscope.
10 Capture videos of the microspheres in three to five different regions of each culture.
11 When saving the video files, adhere to the file naming convention below. This will ensure compatibility with the MATLAB code for tracking described in this manuscript. An example file name for one of the video microscopy files of microspheres undergoing MCT on a culture named “culture A” is as follows: CultureA1_1.
  The underscore is a spacer that is important in helping the MATLAB code recognize the total number of videos being analyzed. It can be changed to another symbol and updated later in the code, if desired. The “1” after the spacer is the total number of videos being analyzed by the code, in this case just one (meaning each video will be analyzed individually and not in a batch). The file for a second video from a second region of the same culture would be named CultureA2_1.
  The code is equipped to run a batch format in which a group of videos can be analyzed using the same parameters. However, it is usually best to analyze videos one at a time to adjust the parameters for each video individually and ensure optimal tracking settings.
12 Repeat steps 9‐11 for other cultures.

#### Collect CBF data

13 After obtaining videos for MCT, switch to the brightfield channel of the microscope to obtain videos of cilia from each culture. Set the exposure time to 20 ms.
  The exposure time and light source intensity can be adjusted if the epithelium appears too dark or too light to see ciliary beating. A higher‐magnification objective may also be used if the ciliary beating is not clearly visible.
14 Focus the microscope to view the epithelium. In some cases, it is possible to see the microspheres traveling on the surface of the mucus in the brightfield videos. This should be avoided by adjusting the *z*‐plane focus.
15 Take a 10‐s video of a randomly selected region of the culture.
16 Save the video file and repeat for three to five total videos in different regions of the same culture.
  There is no specific file naming convention that must be followed for CBF analysis.

#### Analyze MCT data 1: Analyze particle tracking data

**Figure 2:**
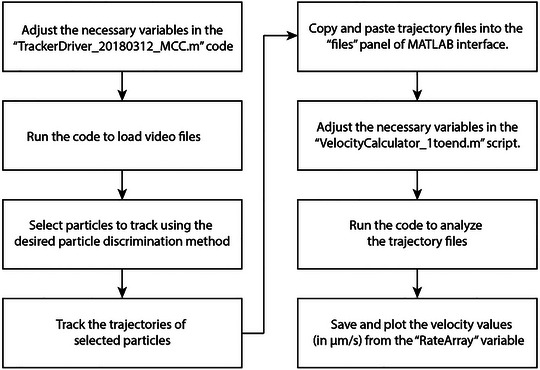
Flow chart of MCT analysis process to obtain microsphere velocity using two different scripts in MATLAB software.

The velocity of MCT is determined by tracking the trajectories of the microsphere particles travelling on the mucosal surface. Particles undergoing MCT are tracked using a MATLAB algorithm that filters out noise in the videos and can then use either of two methods to identify particle centroids (Boboltz et al., 2023; Iverson et al., 2022; Song et al., 2021, 2022). Both methods are based on the original Crocker and Grier algorithm for particle tracking using video microscopy (Crocker & Grier, 1996). The method of particle identification explained in this protocol is most closely based on the MATLAB particle tracking code developed by Daniel Blair and Eric Dufresne, which identifies the brightest pixels and the approximate diameter (in pixels) of the particles used to find particle centroids. Note that this code should only be used to track spherical particles. See Figure 2 for an overview of the MCT analysis process.

TrackerDriver_20180312_MCC.m is the script used to identify and track particles. This script calls multiple functions contained in various accessory MATLAB scripts and Java archive files to load and analyze microscope video files. The installation of a folder containing these files only needs to be done once prior to running the TrackerDriver_20180312_MCC.m code for the first time. In addition, certain add‐on toolboxes available from MathWorks must be installed into the MATLAB software prior to running the script for the first time.

##### Install necessary supporting files and add‐on toolboxes to run the script

17 Download the Particle Tracking Code ZIP file with the folder corresponding to the computer being used (PC or Mac). This folder contains the TrackerDriver_20180312_MCC.m script as well as all the various supporting files needed to run the script. Drag and drop the folder to the desired location on the computer.
18 If processing CZI files (produced by Zeiss microscopes), the bioformats.jar must be added to the static path in MATLAB. Bio‐Formats is a Java library for reading microscope image file formats across various software platforms. To add bioformats.jar to the static path, open MATLAB and enter the following lines of code into the command window to open a blank TXT file in MATLAB:
  cd (prefdir), then enter
  edit javaclasspath.txt
19 In this file, enter the location of the bioformats_package.jar using the following notation:
  C:\Users\your‐username\folder‐with‐the‐code\Particle Tracking code\bioformats_package.jar (for PC)
  /Users/your‐username/folder‐with‐the‐code/Particle Tracking code/bioformats_package.jar (for Mac)
20 Save and close the TXT file, then restart MATLAB.
21 If using the most recent version of MATLAB (version 2025a), both the Image Processing Toolbox and the Statistics and Machine Learning Toolbox available through MathWorks also need to be installed as MATLAB add‐ons before the TrackerDriver_20180312_MCC.m script can be run. In MATLAB, open the “add‐ons” menu and select “explore add‐ons” to search for the necessary toolboxes and install them.

###### Measure mean squared displacement (MSD) and trajectories of particles

22 Open the TrackerDriver_20180312_MCC.m code file in MATLAB. Ensure that MATLAB can access the Particle Tracking Code folder with all its various files. If not, change the current folder or add the folder to the current path.
23 Set the “comp” variable to either “pc” or “mac”.
24 Enter the directory location of the video file you wish to track in the “dir” variable.
  The file location can be found by right‐clicking and selecting “properties” on a PC or “Get Info” on a Mac. Ensure that the “ext” variable has the correct file extension (.tif, .mov, .czi).
25 Enter the file name into the “groupinfo(1).group variable”, which includes everything except the spacer and the number of movies.
  For example, if the full file name is CultureA1_1.tif, the name entered into the variable should be “CultureA1”, because the spacer is the underscore and the 1 after the underscore is the number of movies being analyzed. Do not add the file extension (.tif, .mov, .czi) to the file name.
26 Ensure that the “groupinfo(1).spacer” variable is set to be either a space (“ ”) or a symbol (e.g., “_”) and that the “groupinfo(1).nMovies” variable is set to the correct number of movies in the group (i.e., “one” if analyzing each video individually).
  The code is equipped to run batches of videos using the same parameters applied to all videos. This is not covered in depth here because it usually does not result in optimal particle identification. If you wish to use the batch method, the file naming convention must be changed slightly. For example, for a sample called Culture A with two total videos to analyze in batch format, the first video would be called CultureA_1 and the second CultureA_2. The value of the “groupinfo(1).nMovies” variable would also be changed to two.
27 Adjust the parameters of the “groupinfo(1).lobject” variable to the approximate diameter (in pixels) of the particles being tracked. The entered value must be an odd number, preferably ≥7 pixels.
  If the approximate particle diameter is not known, it can be estimated by opening the video and roughly measuring the particle size in pixels using Fiji software. It is better to overestimate than to underestimate the size.
28 You may change the value of the “groupinfo(1).maxdisp” variable. This variable sets the maximum distance (in pixels) that particles can be displaced between two consecutive frames and must be less than the mean spacing between particles. There is a check implemented later in the process to ensure that this variable is set to an appropriate value (see step 38e).
29 Enter the frame numbers of the first and last frames to be tracked in the “groupinfo(1).startframe” and “groupinfo(1).stopframe” variables, respectively.
30 The next set of variables is used to generate histogram data of the log_10_MSD for all tracked particles, which will be exported as CSV files. Setting the “groupinfo(1).generateLogMSD” variable to 1 will generate histogram data and setting it to 0 will turn this function off. The bins may be adjusted using the “groupinfo(1).minBin” (minimum bin value), “groupinfo(1).maxBin” (maximum bin value), and “groupinfo(1).binsize” (size of each bin) variables. The “groupinfo(1).tau” variable is the characteristic time in seconds. Setting the desired characteristic time will yield final histogram data given as the percentage of particles with various MSD values occurring at the selected characteristic time. After the code finishes running, the exported histogram data for the example file CultureA1_1 will be output in the same location as the original video file with the name CultureA1 ‐ 1‐MSDhist.csv. The CSV file will have two columns of data (see Table 1): the bin values (log_10_[MSD] at the characteristic time) in column A and the percentage of particles in that range at the selected characteristic time in column B.

**Table 1:** Output Guide for CSV File Types Produced by TrackerDriver_20180312_MCC.m

File name convention for video file CultureA1_1	Output CSV file	Output columns						
		A	B	C	D	E	F	G
CultureA1 ‐ 1‐MSDhist.csv	MSD histogram	Bin value, log_10_MSD at *t* = *τ*	% of particles					
CultureA1 ‐ 1‐SNR.csv	Signal/noise ratio	Frame #	Signal/noise ratio					
CultureA1 ‐ 1‐MSD.csv	MSD	Time (s)	Mean MSD	Geometric mean MSD	Median MSD	NaN	MSD particle 1	MSD particle 2…
CultureA1 ‐ 1‐MSDx.csv	MSD (1D in *x*)	Time (s)	Mean MSDx	Geometric mean MSDx	Median MSDx	NaN	MSDx particle 1	MSDx particle 2…
CultureA1 ‐ 1‐MSDy.csv	MSD (1D in *y*)	Time (s)	Mean MSDy	Geometric mean MSDy	Median MSDy	NaN	MSDy particle 1	MSDy particle 2…
CultureA1 ‐ 1‐Trajectories.csv	Particle trajectories	Particle #	Frame #	*x* position (pixels)	*y* position (pixels)			
CultureA1 ‐ 1‐TrajGroup.csv	Particle trajectories (grouped)	Particle #	Frame #	*x* position (pixels)	*y* position (pixels)	Brightness	Radius of gyration	Eccentricity

**Figure 3:**
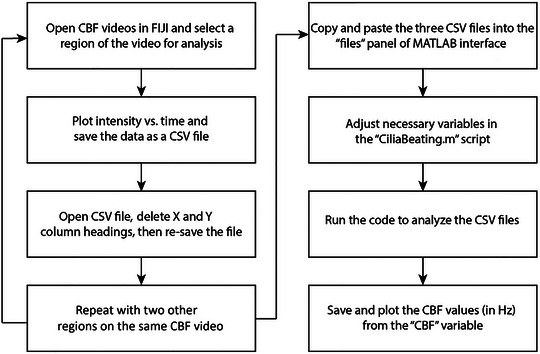
Flow chart of CBF analysis process to obtain ciliary beat frequency using Fiji and MATLAB software interfaces.

**Figure 4:**
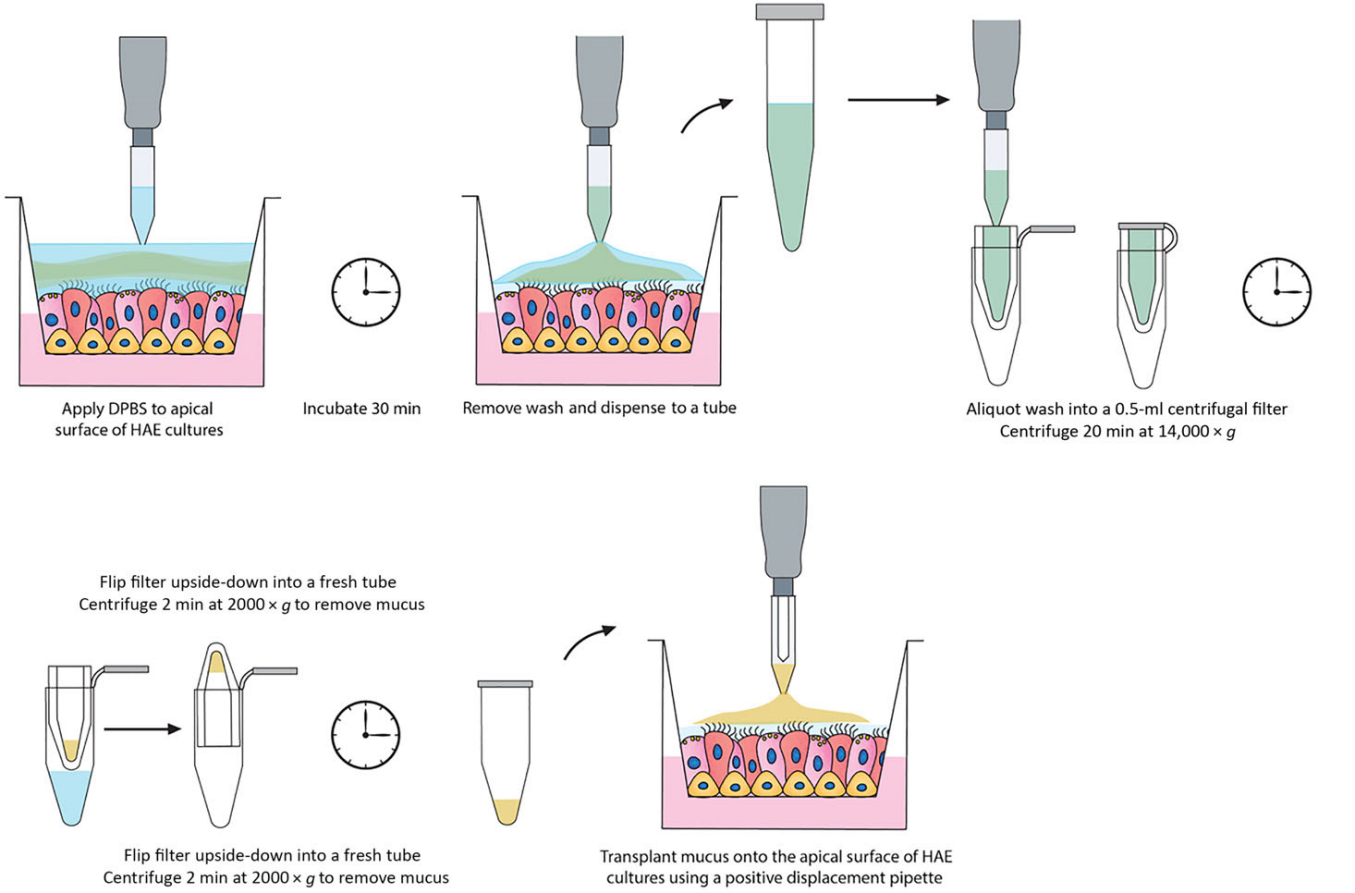
Schematic of procedures for harvesting and transplanting mucus using HAE cultures. In Basic Protocol 2, a 30‐min DPBS wash containing mucus is prepared from a culture and concentrated mucus is isolated using a centrifugal filter. In Basic Protocol 3, the mucus is transplanted onto a culture.

**Figure 5:**
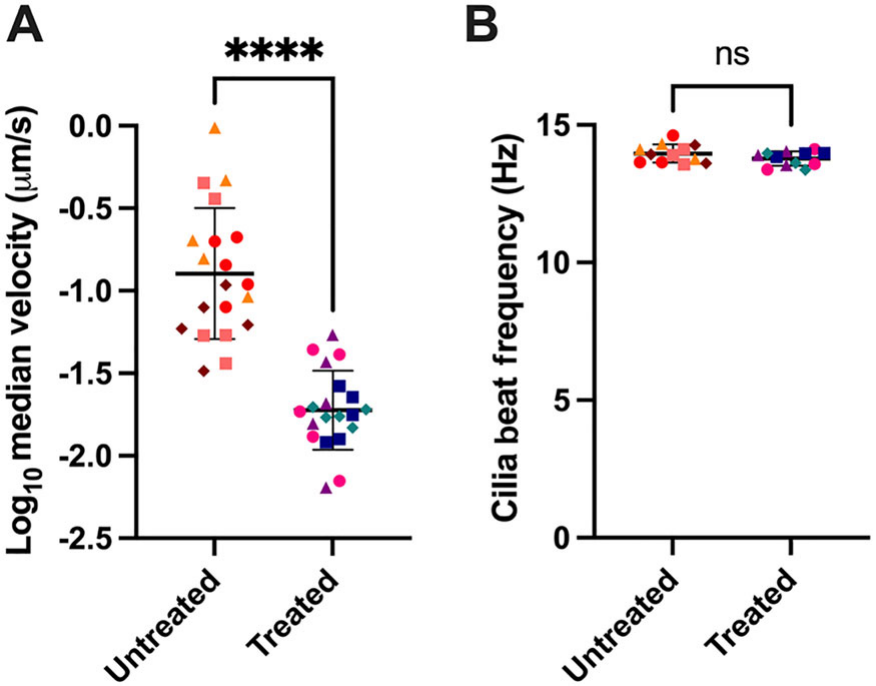
Example data for MCT and CBF comparing untreated BCi‐NS1.1 cultures to cultures treated with an investigational agent. (**A**) Log_10_ of median velocities of tracked microspheres from each video (*n* = 4 cultures per treatment group; *n* = 5 videos in different regions per culture). (**B**) Mean CBF from averaging three different regions analyzed from each video (*n* = 4 cultures per treatment group; *n* = 3 videos per culture). Data point shapes and colors indicate MCT and CBF values from the same culture. Experimental groups were compared using an unpaired two‐tailed *t*‐test (****p < .0001, ns = *p* > .05).

31 Enter the frame rate (in Hz) of the video in the “fpers_user” variable.
32 The “memory_nframes” and “min_frames” variables may be adjusted to determine how long a particle must be in frame in order to track it. The “memory_nframes” variable sets a number of frames that a particle can disappear out of view and still be tracked as the same particle if it comes back into frame. The “min_frames” variable sets a threshold for how many frames a particle must appear in to be tracked. Particles that appear in fewer than this minimum number of frames will be eliminated from the data set and not be tracked.
33 The code uses a bandpass filter on the raw videos, set by the “lnoise” variable. The baseline value is 1 and it is very unlikely that this should be adjusted.
  The code is equipped to run several different particle discrimination methods to identify particles for tracking. The method described here is method 2, which is a thresholding method based on brightness. The particle discrimination method may be changed using the “particleDiscriminationMethod” variable. Method 1 is more sensitive for identifying particles and excluding noise, but requires more user input and thus is a slower data analysis process. Method 1 uses brightness, radius of gyration, and eccentricity to identify particles, and is described below (see steps 40‐44). If method 2 fails to correctly identify particles based on brightness, or if it is important to have the utmost sensitivity in identifying particles, method 1 should be used instead. Method 3 is a batch version of method 1 that uses the same parameters of particle mass, radius, and eccentricity but applies them to multiple videos instead of just one. The “cut_percentile” variable should only be evaluated if the particle discrimination method is set to method 1 or 3 (see step 40 for more information).
34 Enter the conversion factor (in µm/pixel) for the microscope model and objective used in the “scope_conv” variable.
35 Run the code using the Run button in the Editor tab of the MATLAB interface. Once the video is loaded, a figure called “Figure 1” will appear. This figure shows the raw and bandpass‐filtered images of various frames taken at intervals throughout the video.
36 “Figure 1” will be accompanied by a prompt in the command window asking how many regions of the video should be excluded. Regions of high autofluorescence, aggregated particles that are indistinguishable from each other, or other conditions that make finding true particles difficult should be excluded. If there are regions of the video that you want to exclude from tracking, type in the number of regions to exclude and hit enter. A figure will be generated of the raw image of the first frame of the video with a prompt in the command window for you to draw a polygon around the region of interest in which particles should not be tracked. If no regions are to be excluded, type 0 and hit enter.
37 The code will identify particles in the video using method 2 (the automated thresholding method), which can be adjusted manually if desired. Two images will appear, called “Figure 1” and “Figure 2”. “Figure 1” shows a panel of raw and bandpass‐filtered images from the first frame of the video and a surface plot of brightness. “Figure 2” shows the raw image and a surface plot of the image, as well as the final thresholded image of the first frame identifying particles. If regions have been excluded, the polygon drawn around the excluded region will be overlaid on all raw and bandpass‐filtered images of frame 1 in both figures. To accept the default automatic thresholding, enter 1 in the command window after the prompt. To change the threshold value manually, type a 0 after this prompt and then enter a value for the threshold in the prompt that follows. Once this value is entered, a figure will appear with the thresholded image corresponding to the manually input threshold of the first frame. There will also be a prompt in the command window to accept the manual threshold value. Enter 1 to accept the manual threshold value or 0 to adjust it again. Once the automatic or manual threshold value has been accepted, the code will begin tracking the particle centroids identified in the video.
  As centroid identification occurs throughout each frame, there are live updates in the command window indicating the frame in which the code is centroid finding every 25 frames. When finished, this will say “tracking done”. In addition, the total number of particle trajectories tracked will be displayed. Once finished, the elapsed time for tracking will be displayed.
38 Once particle finding and tracking is finished, several output graphs will be generated. There will be a total of three JPG figures, seven CSV files, and one TXT file for each individual video run. All output figures and files will be uploaded in the directory location provided in the “dir” variable (the same location as the original video file). It should be noted that each time the TrackerDriver_20180312_MCC.m code is run for the same file, it will overwrite the files generated from the previous run. Five figures will also appear within the MATLAB interface once the code is finished running.
  a. “Figure 1”: Approximate signal‐to‐noise ratio. This ensures that there is a high‐enough particle signal throughout all frames and should have a value above 0. The information in this figure is also output in the original directory location as a CSV file, where column A is the frame number and column B is the signal‐to‐noise ratio (Table 1). The naming convention for the CSV file for a video titled CultureA1_1 is CultureA1 ‐ 1‐SNR.csv.
  b. “Figure 2”: Pixel bias check. This is a histogram of particle centroid positions within a single pixel to determine if the subpixel resolution is acceptable. The code will count the number of particle centroids per tenth of a pixel, with the summary data shown in the histogram. If the particles are moving, the histograms should be relatively flat. If the particles are moving relatively far and the histogram has a V shape with most of the particle centroid positions being at 0 or 1, consider increasing the diameter of the particles using the “groupinfo(1).lobject” variable and running the code again. Note, however, that if the set size of the particles is too large, there is a risk that bright pixels from nearby particles may generate incorrect particle center positions. Videos with few particles or with many stuck particles will generate histograms that are much noisier. This figure is saved as a JPG file in the original directory location with the naming convention CultureA1_1 ‐ pixel bias check.jpg.
  c. “Figure 3”: Graph of number of displacements over time. This is not automatically output/saved in the directory location.
  d. “Figure 4”: Ensemble average MSD of all tracked particles on both log and linear scales. Each graph shows the ensemble average MSD over time, including the MSD in either the *x* or *y* plane alone. Both plots are automatically output in the directory location as JPG files. For the video file CultureA1_1, the name will be CultureA1 ‐ MSD plot.jpg. In addition, the MSD values for each particle tracked in the video are output in three CSV files in the directory location: MSD, MSDx, and MSDy (Table 1). The naming convention is CultureA1 ‐ 1‐MSD.csv, CultureA1 ‐ 1‐MSDx.csv, and CultureA1 ‐ 1‐MSDy.csv. In addition, if the code is set to output a histogram of MSD values (see step 6), this histogram will be output under the filename CultureA1 ‐ 1‐MSDhist.csv in the same directory location. The MSDs can be extracted from the MSD CSV files and plotted. To read the MSD, MSDx, or MSDy CSV files, the file is structured as follows:
    Column A = Time (s)
    Column B = Mean MSD
    Column C = Geometric Mean MSD
    Column D = Median MSD
    Column E = A spacer column with no data (to separate summary data from data obtained from each individual particle (NaN)
    Columns F, G… = MSD (one column for each particle)
  e. “Figure 5”: Histogram of displacements at the shortest timescale. Prior to analyzing the next video, check that the maximum displacement value of a particle set in the “groupinfo(1).maxdisp” variable is large enough. The histogram should taper down to a count of almost 0 below the max displacement value. If values within this histogram are biased toward the higher end of the *x* axis, increase the value of this variable to ensure that particles traveling at high speeds are tracked effectively. Do not go too high, as this will slow the running speed. This histogram will be automatically output in the directory location as a JPG file with the naming convention CultureA1 ‐ maxdisp check.jpg.
  f. A TXT file called “Tracking Info” is also output in the directory location that contains information on the parameters used to analyze each video and the total number of trajectories tracked. The naming convention is CultureA1 ‐ 1 ‐ TrackingInfo.txt.
  g. The trajectories and trajgroup CSV files contain information about the specific *x* and *y* positions of each particle during each frame of the video. These files are automatically saved to the directory location. The naming convention for these files is CultureA1 ‐ 1‐Trajectories.csv and CultureA1 ‐ 1‐TrajGroup.csv (Table 1). The structure of these CSV files can be read as follows:
*For trajectories files*:
    Column A = particle identifier number assigned by the code
    Column B = video frame number
    Column C = *x* position (in pixels)
    Column D = *y* position (in pixels)
*For trajgroup files*:
    Column A = particle identifier number
    Column B = video frame number
    Column C = *x* position (in pixels)
    Column D = *y* position (in pixels)
    Column E = brightness
    Column F = radius of gyration
    Column G = eccentricity

In trajectory files, all particles start at position (0, 0). In trajgroup files they do not.

39 It is possible to calculate the velocity of MCT for each identified particle based on the distance the particle travels over time in the trajectories CSV file. To do this, proceed to step 45.

#### Analyze MCT data 2 (optional): Using an alternate particle discrimination method

The particle discrimination method used in the TrackerDriver_20180312_MCC.m code may be changed using the “particleDiscriminationMethod” variable to method 1 or method 3. Whereas method 2 uses a brightness threshold and the input particle size to determine which spots are true particles and where the centroids are, method 1 identifies particles based on particle radius of gyration, brightness, and eccentricity. Method 3 is a batch version of method 1, using the same parameters but applying them across multiple videos.

40 Assess the value of the “cut_percentile” variable. Spots with a brightness below the cut percentile will be excluded and those with a brightness above this value will be assessed further to determine if they are particles. It is most important that the cut percentile be set low enough to detect the particles of interest. If the cut percentile is too low, however, the code will waste time identifying the centroids of noise spots. Often, using a value of 0.05 (which will show spots that fall only within the top 5% of brightness for all pixels in the video) is more than enough to correctly identify particles; it can likely be lowered to 0.03 or even 0.01.
41 When the code begins running, a figure will appear that shows raw images of various frames taken at intervals throughout the video and a prompt will appear in the command window asking how many regions of the video should be excluded. If no regions are to be excluded, enter 0. See step 36 if regions need to be excluded.
42 The code will identify the centroids of spots that are potentially particles. The frame being analyzed will be displayed in the command window and a figure called “Figure 1” will appear showing a colorful graph of the radius of gyration squared (a measure of particle size) versus brightness. The data points represent centroids and their colors correspond to the frame the centroids are from in order to help identify potential photobleaching (blue is the first frames and red is the last frames). The command window will prompt you to draw a polygon around the data points on the graph that you want to keep as potential particles. Draw the polygon with the mouse.
  The goal is to exclude noise and aggregate particles. There is often a large number of dim red centroids forming a large vertical‐shaped cluster very close to the y axis. This is usually noise and should be excluded. Normally, it is best to exclude data points from late in the video far toward the y axis.
43 Once the polygon shape is closed, the code will generate two more figures. “Figure 2” is an image of the particles identified in the last frame of the video according to the selected centroid data points within the polygon. It will have an overlay of open circles in red showing what the code identified as a particle. “Figure 3” is a graph of eccentricity versus brightness. The command window will prompt you again to draw a polygon around the centroid data points on the graph that you want to keep. A recommendation is to exclude centroids >0.3 (i.e., draw the polygon around centroids ≤0.3). Once this selection has been made, “Figure 4” will appear showing an image of the particles identified, outlined in green, in the last frame of the video according to the selection made on the eccentricity versus brightness graph. A prompt will appear in the command window for you to type 1 if satisfied with the identified particles or 0 if unsatisfied. If you enter 0, the process of drawing polygons on the graphs will begin again. If the red and green circles drawn around the outline of the spots identified as particles in “Figures 2 and 4” are much too large or small compared to the size of the fluorescence particles in the image, some optimization can be done by choosing a better approximate particle size (in pixels) in the “groupinfo(1).lobject” variable. If particles are not being identified accurately, it may also be worthwhile to revisit the cut percentile to ensure that it is not too high and is excluding particles.
44 Once you are satisfied with the particle discrimination, the code will begin tracking the identified particles. The number of frames tracked will update in real‐time in the command window. The output files and locations are the same as with method 2 described above. To calculate microsphere velocity, proceed to step 45.

#### Analyze MCT data 3: Calculate microsphere velocity

The velocity of MCT of the tracked microspheres can be determined after using any of the particle discrimination methods within the TrackerDriver_20180312_MCC.m code.

45 Open the VelocityCalculator_1toend.m MATLAB code.
46 Copy and paste the “Trajectories” CSV file produced by the TrackerDriver_20180312_MCC.m code into the side bar of MATLAB. This will be in the “files” panel of the interface for newer MATLAB versions or in the “current folder” panel for older versions.
47 Enter the complete file name in the “TrajFile” variable. For example, the file name produced for the video CultureA1_1 would be CultureA1 ‐ 1‐Trajectories.csv.
48 Enter the exposure time (in s) in the “time” variable.
49 Enter the total number of frames in which particles were tracked in the “numFrame” variable.
50 Run the code using the run button in the editor tab of the MATLAB interface. This will produce several variables in the “workspace” sidebar of the MATLAB interface when run. To obtain the velocity of all particles from the trajectories produced in a particular video, double‐click to open the “RateArray” variable in the workspace window. This variable should be a single‐column variable that has the velocities of each particle tracked in a single video (in µm/s). The total number of values in the “RateArray” variable should be the same as the total number of trajectories tracked for that video. Copy the entire column of velocities and paste it into another program (e.g., Microsoft Excel or GraphPad Prism) to save these values. Each time a new “Trajectories” CSV file is run, the variable will change. The VelocityCalculator_1toend code does not save velocity values from previous files.

#### Analyze CBF data

To quantify the ciliary beat frequency based on videos taken using brightfield microscopy, multiple regions of each of the videos will first be selected and processed in Fiji. The beating of cilia is identified and quantified by Fiji based on the number of local pixel intensity maxima over time. After saving the pixel intensity data from Fiji, it is run in a MATLAB code that divides the number of local pixel intensity maxima or “beats” over the total time of the video to compute CBF (Boboltz et al., 2023; Iverson et al., 2022; Song et al., 2021; Song et al., 2022). See Figure 3 for a summary of the CBF analysis process.

51 Open a brightfield video of the epithelium in Fiji. Select the circle tool in the toolbar and create a circle of random size in a random region of the video.
52 Select Image/Stacks/Plot *z* axis profile. This should yield a graph of the mean intensity versus time (in s). The mean intensity (*y* values) should oscillate rapidly over time, indicating that ciliary beating was detected in that region.
53 At the bottom of this graph, select “data” and click “save data” to save the mean intensity versus time data as a CSV file.
54 Repeat the selection of random regions and plot the *z* axis profile two more times to give a total of three CSV files from three different regions of one video.
55 Each CSV file will have two columns labeled “X” and “Y” in columns A and B of the file corresponding to the data from either the *x* or *y* axis of the intensity versus time graph from which it was saved in Fiji. Column A (labeled X) will be the elapsed time of the video. Column B (labeled Y) will be the mean intensity at that time point. The MATLAB code is not able to read the X and Y lines, only the numbers underneath. Thus, open the CSV file from each region, delete the “X” and “Y” headings in the first rows of Columns A and B, and re‐save the file.
56 Open the MATLAB script CiliaBeating.m.
57 Copy and paste the CSV files to the “files” side panel (newer MATLAB versions) or “current folder” side panel (older versions).
58 Enter the names of the CSV files into the “IntensFile”, “IntensFile2”, and “IntensFile3” variables.
59 Run the code. This will generate three intensity versus time plots (“Figures 1‐3”) in which the local maxima (indicating a ciliary beat) are highlighted with red asterisks. Open the CBF variable by double‐clicking the variable in the workspace sidebar. Copy and paste the three values (corresponding to each region of the video) into a separate program (e.g., Excel or Prism) to save them, because each time the code is run with different CSV files the values within the array will be changed.
60 Repeat this process for all videos taken for each culture, compiling the data in a plotting software such as Prism. If three videos are taken for one culture, there should be a total of nine CBF values from nine different regions (three regions per video).

## Basic Protocol 2 COLLECTION OF MUCUS FROM HAE CULTURES

Mucus is secreted by a variety of immortalized and primary airway epithelial cell lines grown at an air‐liquid interface on cell culture membrane inserts. The protocol for collecting this mucus is split into two sections: (1) collecting a mucus‐containing wash from the mucosal surface of HAE cultures and (2) isolating concentrated mucus from the wash using a centrifugal filter. Figure 4 shows an overview of these procedures.

### Additional Materials (also see Basic Protocol 1)

Sterile 15‐ml conical tubes or other sterile container
Amicon Ultra 0.5‐ml, 100‐kDa cutoff centrifugal filters (Millipore‐Sigma, cat. no. UFC5100)

#### Harvest mucus

1 Prepare the BSC for sterile cell culture work, wiping any materials brought into the hood with 70% isopropanol or ethanol.
2 Prepare a 15‐ml conical tube containing enough sterile DPBS to wash each culture with 250 µl. Warm in a 37°C water bath for at least 15 min, then place in the BSC.
3 Transfer HAE cultures from the incubator to the BSC.
4 Gently dispense 250 µl sterile DPBS onto the apical surface of each culture, making sure not to touch the epithelial cells.
5 Place the lid on the plate and return to the incubator for 30 min.
6 Return the plate to the BSC.
7 Use a 1000‐µl pipette tip to draw up the DPBS wash (containing mucus) from the first culture, leaving very little or no DPBS on the surface. Dispense into an appropriate sterile tube to hold the volume of wash.
  The size of the tube will depend on the number of cultures and the experimental design. The DPBS washes from all cultures can be pooled into a single container or they can be dispensed into separate containers for individual cultures or treatment groups.
  When removing the DPBS, gently tip the plate forward with one hand so the DPBS pools toward the edge of the apical compartment of the Transwell but does not leak out into the basolateral compartment. Be careful not to touch the cell culture surface with the tip of the pipette.
  If there is a large amount of mucus or the mucus is thick, a “string” of mucus may come off the apical surface of the culture when pulling the pipette tip up and away. The DPBS wash in the tube should look slightly cloudy and there may be stringy particulates that precipitate at the bottom of the container.
8 Repeat for other cultures, dispensing into pooled or individual tubes as needed.
9 Place the lid back on the plate and return cultures to the incubator.

#### Isolate mucus by centrifugation

10 Label microcentrifuge tubes (provided with centrifugal filters) with sample identification information, date of collection, number of days the cultures have been maintained at the air‐liquid interface, and any other pertinent details.
11 Place centrifugal filters into the labeled tubes according to manufacturer's instructions.
12 Add 250‐500 µl DPBS wash onto a filter using a 1000‐µl pipette and close the lid over the filter.
13 For wash volumes >500 µl, repeat with additional centrifugal filters.
14 Centrifuge samples for 20 min at 14,000 × *g*.
  Larger‐volume 100‐kDa centrifugal filters may be used, but the centrifugation steps may need to be adjusted according to the manufacturer's recommendations. For instance, if using 15‐ml, 100‐kDa centrifugal filters, the pooled washes should be centrifuged for 40 min at 4000 × g.
  After centrifugation is complete, a large volume of flowthrough will have collected in the microcentrifuge tube and the mucus will be concentrated inside the filter.
15 While samples are spinning, label a new set of microcentrifuge tubes as above.
16 Remove the filter from the tube and flip it upside‐down into a new labeled tube so the opening of the filter is facing down into the new tube (see manufacturer's instructions). Discard the old tubes containing flowthrough.
  The lids of the tubes will remain open when the filters are upside‐down in them (Fig. 4).
17 Place the tubes in the microcentrifuge with the lids facing toward the center of the rotor. Spin for 2 min at 2000 × *g* to remove the mucus from the inside of the filter.
  There should now be a small volume of mucus in the bottom of the centrifuge tube. Each culture typically yields ∼30‐70 µl mucus, but this may vary significantly depending on the cells used.
  For larger‐volume centrifugal filters, collection of concentrated mucus from the filter requires manual removal using a positive‐displacement pipette with sterile disposable capillary piston tips.
18 Remove filters from the tubes and close the lids. Store mucus up to ∼2 weeks at 4°C or long‐term at −80°C. Avoid multiple freeze‐thaw cycles.

## Basic Protocol 3 TRANSPLANTATION OF MUCUS TO HAE CULTURES AND INFECTION WITH VIRUS

This protocol is used to transplant mucus from various sources to fully differentiated HAE cultures. Cultures are first washed to remove native mucus along with any cell debris. A mucus sample (along with other mucus samples of interest with matched volumes) is then added to the apical surface of the culture. The culture is incubated at 37°C to allow the transplanted mucus to equilibrate and spread evenly across the culture, facilitated by mucociliary transport. Virus inoculation is then performed to assess the barrier function of the transplanted mucus against infection by respiratory viruses in the HAE model. Virus inoculum is added apically at small volumes to avoid disrupting the transplanted barrier and is left for set times, allowing viruses to navigate through to underlying cells.

### Additional Materials (also see Basic Protocol 1)

Concentrated mucus samples (see Basic Protocol 2)
Complete ALI medium: PneumaCult‐ALI basal medium with PneumaCult‐ALI supplement and PneumaCult‐ALI maintenance supplements (StemCell Technologies, cat. no. 05001) plus 1% penicillin/streptomycin (Gibco, cat. no. 15140‐122)
Virus of interest with known titer
Aqueous 8% (w/v) paraformaldehyde, EM grade (EMS, cat. no. 157‐8)

Sterile 50‐ml conical tubes
Sterile 1.5‐ml tubes
Positive‐displacement pipette for volumes up to 100 µl
Sterile disposable capillary piston tips for positive‐displacement pipette

#### Prepare mucus and wash cultures

1 If mucus samples were frozen at −80°C, allow to thaw overnight at 4°C.
  Thawing too quickly at 37°C may result in sample degradation and unwanted changes to the viscoelastic properties of the mucus.
2 Prepare the BSC for sterile cell culture work, wiping any materials brought into the hood with 70% isopropanol or ethanol.
3 Prepare one 50‐ml conical tube with enough DPBS for 250 µl per culture and a second 50‐ml tube with enough complete ALI medium for 1 ml per culture. Place in a 37°C water bath for at least 15 min and then place in the BSC.
4 Transfer HAE cultures from the incubator to the BSC.
5 Rinse cells by aspirating the basolateral medium from each culture and replacing with 1 ml warm complete ALI medium.
6 Gently dispense 250 µl sterile DPBS onto the apical surface of each culture, making sure not to touch the epithelial cells.
7 Place the lid on the plate and return to the incubator for 30 min.
8 Return the plate to the BSC and carefully aspirate off as much of the DPBS as possible using an aspirating pipette and vacuum.
  This removes much of the secreted mucus as well as cell debris. The procedure is the same as for harvesting mucus (see Basic Protocol 2), except that there is no need to collect the wash. Tilt the plate to pool the DPBS before aspirating with a 200‐µl non‐barrier tip, and be careful not to not touch the cells with the tip.

#### Transplant mucus

9 Use a positive‐displacement pipette with corresponding sterile capillary piston tips to carefully pipette 20 µl thawed mucus sample to the apical surface of each culture, being careful to avoid touching the cultures with the pipette.
  This volume is calculated to yield a ∼50 µm barrier in a 12‐well HAE culture.
10 Gently rock the plate and then place in the incubator for 50 min to allow the mucus to spread and equilibrate.
  The mucus needs time to sufficiently spread across the surface of the culture. The motion of the cilia will aid in this process.

#### Inoculate cultures

11 While the transplanted mucus is equilibrating, dilute the virus in DPBS as needed for infection.
  The volume of virus added to each culture should not exceed 4 µl to ensure that it does not cause the transplanted mucus to swell and change its microarchitecture. For high‐titer virus samples, dilute to achieve the desired titer in 4 µl. As an example, in an influenza A virus (IAV) infection, we typically dilute our stock to achieve a multiplicity of infection (MOI) of 0.1 in 4 µl, estimating 1.5 × 10^5^ target cells at the apical surface for infection.
12 At the end of the equilibration period, transfer cultures to the BSC.
13 Inoculate the culture surface with 4 µl prepared virus. Administer the virus‐containing droplet to the center of the culture (it will spread during incubation), being careful not to disrupt the transplanted mucus barrier.
14 Return to the 37°C incubator and incubate for at least 15 min to allow the virus to adsorb.
  Longer inoculation times may be utilized depending on the experimental goals and/or specific virus used. This time is optimized for our experiments with IAV. Care should be taken to ensure proper preliminary studies have been done to determine the optimal inoculation time as well as the total infection time.
15 Remove inoculum from cultures by adding 250 µl warm DPBS and quickly aspirating.
  Removing the DPBS quickly ensures that the wash does not impact virus movement though the mucus barrier and that any non‐adsorbed virus and transplanted mucus are removed rapidly.
16 Wash a second time by adding 250 µl DPBS and incubating at 37°C for 15 min before aspirating.
17 Return cultures to the incubator at 37°C for the duration of the infection period.
  For IAV, we typically allow infection to proceed for 12‐24 hr.
  Lower temperatures reflective of the proximal airways may be used for viruses that preferentially target the upper respiratory tract.

#### Prepare apical washes and fixed cultures for analysis

After infection is complete, apical washes can be used to determine the infectious viral titer or viral genome copy number across time points and conditions. Additionally, cultures can be fixed and stained *en face* according to protocols developed for visualizing specific viral and cellular components (Gagliardi et al., 2022).

18 Add 200 µl warm DPBS apically to each culture and incubate for 30 min at 37°C.
19 During incubation, prepare 4% paraformaldehyde in DPBS by mixing equal volumes of 8% paraformaldehyde and DPBS to yield a total volume of 1.5 ml per culture.
20 Collect apical washes individually into sterile 1.5‐ml tubes.
  Again, tilt the plate toward you to allow the DPBS to pool to the side, and do not allow the pipette tip to touch the cells. Collect as much of the wash as possible.
21 Freeze washes immediately at −80°C and store until analysis (can be stored indefinitely).
22 Aspirate and discard all basolateral medium and any residual apical DPBS.
23 Add 500 µl of 4% paraformaldehyde to the apical surface and 1 ml to the basolateral side. Incubate for 15 min at room temperature to fix the sample.
24 Remove paraformaldehyde. Wash by adding 500 µl DPBS apically and 1 ml basolaterally and removing by aspiration.
25 Add DPBS again, apically and basolaterally, and store at 4°C until analysis (can be stored indefinitely).
  Ensure that the DPBS does not dry out before analysis can be performed.

## COMMENTARY

### Critical Parameters and Troubleshooting

#### MCT and CBF

The MCT velocity and CBF will likely be dependent on culture conditions as well as the HAE cells used. For example, primary HAE cells may have different MCT rates and CBF values than immortalized BCi‐NS1.1 HAE cells grown at the ALI. The MCT velocity may also be dependent on factors such as the region of the culture being imaged. Because mucus often accumulates around the outer edges of the circular cell culture insert, microsphere transport in this region may differ from the center of the insert, where the mucus layer is likely thinner. The frequency with which HAE cultures are washed to remove mucus as part of normal culture maintenance will also likely affect MCT rate and should be considered before beginning an MCT experiment. The length of time that microspheres are left on cultures is also an important parameter. Different microsphere incubation times can lead to variable results in velocity, likely due to changes in the hydration of the mucus gel layer as residual PBS is absorbed. It is possible to leave the microspheres on the cultures overnight prior to data collection, but the microspheres may move more slowly or be largely immobile.

The imaging protocol described above is sufficient to detect changes in the CBF. However, it is important to note that CBF data collection and quantification protocols have limitations and can be further refined to improve the accuracy of quantification. For example, alterations in imaging conditions between videos can lead to differences in background noise levels that can affect measurement of CBF. Therefore, it is important to keep the imaging conditions as consistent as possible. If there is excess background noise (i.e., smaller local maxima and minima that do not appear to be associated with ciliary movement), or if there are large differences in the amount of background noise between videos, it may be useful to adjust the Gaussian smoothing variable (default value of 0.2) in the MATLAB script. The CiliaBeating.m code is automatically to set minimal smoothing of the intensity versus time data obtained from the CBF videos in Fiji. Increasing the smoothing values will reduce the smaller fluctuations associated in mean pixel intensity due to noise. Furthermore, adjusting the imaging settings to take videos at a higher magnification (20‐63x) and at a frame rate exceeding the recommended 50 fps in this protocol may improve the accuracy of CBF quantification. According to previous research, 50 fps is sufficient to capture ciliary beating in airway epithelial cultures but may be improved with a higher frame rate (Scopulovic et al., 2022).

For troubleshooting the data collection and analysis for MCT, see Table 2.

**Table 2:** Troubleshooting for MCT Analysis

**Problem**	**Possible cause**	**Solution**
No transport of microspheres on HAE cultures	The mucus layer of the HAE culture is not optimally hydrated for normal MCT	Shorten incubation time after application of microspheres and/or increase microsphere volume applied
	HAE cells are not viable or are not fully differentiated (i.e., inadequate number of ciliated cells)	Check the trans‐epithelial electrical resistance (TEER) of the HAE culture, inspect by brightfield microscopy, or check for presence of ciliated and secretory cells using immunofluorescence
Microspheres are clumped together and indistinguishable from one another	Imaging is focused on the outer edges of the culture insert where there is thick mucus buildup	Move to a different region of the culture and/or exclude areas with significant microsphere clumping during MATLAB analysis
	Microspheres are not sufficiently diluted or are aggregated	Dilute microspheres further in DPBS and/or sonicate microspheres again
	Incubation time is too short for microspheres to spread over the mucosal surface	Increase incubation time to allow microspheres to distribute more evenly
	Microscope settings are not optimal	Reduce exposure time and/or laser intensity so individual microspheres are distinguishable
TrackerDriver_20180312_MCC.m dode is giving the error “Unable to recognize function or variable ‘loadMovie_dissertation’”	The video file is not able to be loaded because the directory location or file name is incorrect	Edit the directory location in the “dir” variable or the video file name in the “groupinfo (1).group” variable

#### Mucus transplantation

The purpose of replacing the native mucus on an HAE culture with transplanted mucus or a synthetic hydrogel is to enable rigorous interrogation of mucus barrier function by controlling the volume and composition of the barrier. In setting up an experiment, the volume of mucus or synthetic hydrogel added should allow for even spread across the culture without disrupting ciliary beating or MCT. Importantly, the characteristics of the transplanted mucus can and should be investigated alongside its barrier function. Understanding physiochemical characteristics including mucin content, percent solids, and pore size of the mucus gel will aid in the identification of pertinent qualities impacting virus movement and subsequent infection. It is important to note that the specific characteristics of mucus from different sources can vary greatly. By using a synthetic hydrogel, one can evaluate the impact of pore size and microarchitecture in the absence of protein components that may impact virus movement through adhesive interactions (e.g., via glycan binding). Previously, we described a PEG‐based synthetic hydrogel barrier that recapitulated the network structure of native mucus produced by BCi‐NS1.1 airway basal cells at the ALI (Kaler et al., 2025).

For troubleshooting mucus transplantation, see Table 3.

**Table 3:** Troubleshooting for Mucus Transplantation and Viral Infection

Cytopathic effects observed in cultures with transplanted mucus	Mucus volume is too high and/or incubation is too long	Reduce volume and/or transplantation time
Infection frequency too low	Low titer of virus stock	Consider concentrating virus stock by ultracentrifugation
	Mucus barrier is too thick or dense	Decrease volume of mucus transplanted and/or increase virus inoculation time to allow more virus to navigate through the transplanted barrier to the underlying cells
Infection frequency too high	MOI is too high	Dilute virus stock further
	Inoculum was left for too long	Shorten the time before the inoculum is rinsed off
	Infection period was too long	Shorten the time before fixing the culture

#### Infection frequency

The amount of infectious virus and the inoculation time and duration of infection should be adjusted to optimize the infection frequency. Ideally, upon fixation, the infection frequency in the no‐mucus control should be ∼50% of total target cells. This establishes a relatively large dynamic range, permitting observation of decreased or increased infection frequency in experimental conditions. For instance, it is more difficult to observe reductions in infection frequency if only a small percentage of cells are infected in the absence of any mucus barrier. For inoculation time, one may consider using a time based on the anticipated clearance rate of inhaled particulate from the airways *in vivo*, which is on the order of 15‐60 min (Lai et al., 2009). Another consideration is the use of antibodies or antivirals (e.g., neuraminidase inhibitors for IAV) after inoculation to facilitate detection of initially infected cells by blocking virus spread.

For troubleshooting viral infection, see Table 3.

### Understanding Results

#### MCT and CBF

Differences in MCT between treatment groups can be assessed by plotting the calculated velocities of the tracked particles after analyzing videos using the TrackerDriver_20180312_MCC.m and VelocityCalculator_1toend.m codes. It is important to ensure that there is measurable transport of the microspheres above 0.05 µm/s in untreated control cultures using microspheres that are 2 µm in diameter. Below this value, it becomes very hard to distinguish differences in MCT because a microsphere moving at this rate would not travel farther than its radius (1 µm) in the 20‐s duration of the video. This would mean that the microsphere would not roll over even once in 20 s and is considered immobile.

Using several replicate HAE cultures for each treatment or control group and recording multiple videos of different regions from each culture can produce hundreds or thousands of velocity values from tracked particles. Often, the median particle velocity for each video of a particular culture is plotted and reported. For example, Figure 5A depicts four replicate cultures per treatment group (treated and untreated) with five videos per culture, yielding a total of twenty median velocity data points per group (4 cultures/treatment × 5 videos/culture). Since the velocity values can span a wide range, plotting the log_10_ of the velocities can make it easier to visualize differences. The CiliaBeating.m code will yield three different CBF values per video (one for each region selected using Fiji). Figure 5B depicts four replicate cultures for each treatment group with three videos per culture. During Fiji analysis, three distinct regions of each video are analyzed, yielding a total of 36 CBF values per culture (4 cultures/treatment × 3 videos/culture × 3 regions/video). Typically, we take the mean of the three CBF values from the three regions of each video to produce a total of twelve values per treatment group.

#### Virus infection

The primary aim of an infection experiment with transplanted mucus or synthetic hydrogels is to evaluate the specific barrier function of the transplanted material in a physiologically relevant *in vitro* HAE model. The data generated from this type of experiment will reveal the relative protective function of the transplanted barriers investigated in the context of the virus of interest. Virus titers can give insight into the infection rates under different conditions. Typically, the more virus particles that successfully penetrate the overlaid barrier to infect the underlying cells, the earlier you can detect infectious progeny in the apical wash and the higher the viral titers will be at later time points. This should also be reflected in viral genome copy numbers in the apical washes as determined by qRT‐PCR analysis.

In analyzing and interpreting these results, it is important to highlight the potential impact of native mucus, which may dilute the impact of a transplanted barrier. This is a complication of using HAE cultures that continue to produce mucus during the course of a transplantation experiment. This aspect of the system should also be considered when selecting the length of time between virus inoculation and the final experimental time point, where shorter time frames are preferred in order to limit the potential impact of endogenous mucus production on the results.

**Figure 6:**
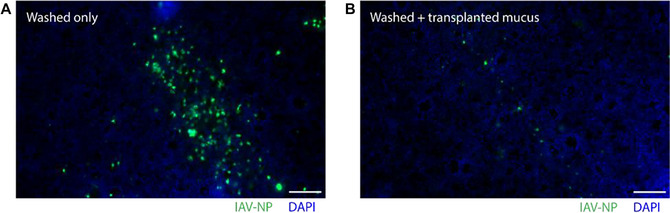
Immunofluorescence detection of IAV nucleoprotein (NP) in HAE cultures with and without a transplanted mucus barrier. Differentiated HAE cultures were washed to remove native mucus and either left without a mucus barrier (**A**) or treated with transplanted mucus (**B**) before inoculation with influenza A/Udorn/307/1972 (H3N2) at MOI = 0.1. Cultures were washed after 1 hr to remove any non‐adsorbed virus. Infection proceeded for 12 hr before cultures were fixed and stained for nuclei (DAPI) and IAV‐NP (1:100 anti‐IAV NP; clones A1, A3 blend; Millipore‐Sigma, cat. no. MAB8251). Scale bar, 100 µm.

Following infection, the fixed culture may be used for immunofluorescence staining for viral antigens in order to visualize infection frequency and spread (Fig. 6). Quantification of infection frequency from *en face* images can be performed by establishing protocols for automated analysis within the software of choice (e.g., ImageJ or CellProfiler) for a set of representative images within a particular virus infection (Gagliardi et al., 2022; Stirling et al., 2021).

### Time Considerations

#### MCT

The time needed to collect MCT and CBF data is highly dependent on the total number of cultures being imaged. The process of preparing the fluorescent microsphere solution, applying it to the cultures, and equilibrating the microspheres on the mucosal surface should take ∼30‐35 min. The microscope can be set up during the 15‐min microsphere equilibration period. Obtaining videos in various regions of each culture should be done relatively quickly to avoid large alterations in MCT or CBF caused when cultures spend long periods outside the incubator. Usually, the goal is to obtain MCT and/or CBF videos for all cultures within 20 min. Videos for CBF may be obtained during the same imaging session as the MCT videos by simply switching the microscope settings.

One recommendation is to work in batches of cultures (usually two to three at a time), where one batch could be all replicate cultures of a single treatment or control group. This batch method may require extra time, as each set of cultures must be moved to a separate 12‐well plate and ushered through all remaining steps of the protocol before the process can be replicated for the next group of cultures. The batch method is not necessary when using a small number of HAE cultures or when using a microscope incubation chamber set to 37°C and 5% CO_2_. In these cases, all HAE cultures can have microspheres applied and be imaged in one session.

The time required for MCT and CBF data analysis is also highly dependent on the number of HAE cultures used in the experiment and the number of videos from each culture. One consideration is that the codes will take longer to run if using a lower exposure time setting on the videos (more frames per second). Timing will also vary based on the method of particle tracking selected in the TrackerDriver_20180312_MCC.m code.

#### Infection

Both the inoculation time and the duration of infection will depend on the particular virus and specific experimental question. The total time—from initial infection to collection of the final apical wash and fixation of cultures—may range from as little as 6 hr for a virus with more rapid replication kinetics to 72 or 96 hr. It is important to consider the initial MOI as well as potential cytopathic effects of the virus on the culture to help determine experimental parameters. For example, a higher MOI and longer infection time course may result in excess damage to the culture, impacting virus titer and/or detection of infection via immunofluorescence.

### Author Contributions

**Maria Corkran**: Data curation; methodology; writing ‐ original draft; writing ‐ review and editing. **Allison Boboltz**: Data curation; methodology; writing ‐ original draft; writing ‐ review and editing. **Gregg A. Duncan**: Conceptualization; writing ‐ review and editing. **Margaret A. Scull**: Conceptualization; writing ‐ review and editing.

### Conflicts of Interest

The authors have no conflicts of interest to declare.

## Data Availability

Data that support the findings of this study are available from the corresponding author upon reasonable request.
